# Infected abdominal aortic aneurysm graft complicated by lumbar discitis

**DOI:** 10.1259/bjrcr.20170101

**Published:** 2018-02-09

**Authors:** Hamzeh B Sumrein, Sally D Parry, Ravi V Ayer, Andrew P Leonard

**Affiliations:** 1 Department of Radiology, Poole Hospital NHS Foundation Trust, Poole, UK; 2 Department of Gastroenterology, Poole Hospital NHS Foundation Trust, Poole, UK

## Abstract

We present the case of a 68-year-old male, who underwent open abdominal aortic graft in August 2016 owing to a ruptured large infrarenal abdominal aneurysm. He subsequently presented 6 months later with back pain, general weakness, reduced mobility and cachexia. He underwent CT, MRI and fluorodeoxyglucose (PDG)-PET spinal imaging, all modalities showing signs of aortic graft infection complicated by L4/5 discitis. The patient was treated conservatively with intravenous antibiotics and spinal brace support, as his general condition did not allow for surgery. Although he showed initial clinical improvement allowing plans for supported discharge, his improvement was not sustained and he died 4 months after admission.

## Clinical presentation

A 68-year-old male presented as an emergency in August 2016 with a diagnosis of a ruptured infrarenal abdominal aortic aneurysm. He underwent emergency open vascular surgery with the placement of an aortic bifurcated graft with intraoperative right femoral embolectomy. One day post-operatively, he required a left femoral embolectomy. In February 2017—6 months after his surgery—he presented through the emergency department with worsening low back pain, progressive fatigue, decreased appetite and oral intake. He was severely cachexic. His notable comorbidities included chronic renal failure and had a past history of excess alcohol intake.

 On admission, he was apyrexial (36.7 C), normotensive (BP 110/70 mmHg) with a normal pulse rate (68 per min). He was severely cachectic weighing 37 kg with a body mass index of 12.4. On palpation of the lumbar spine, there was tenderness without radiation and there were no focal neurological signs in the lower limbs. His breathing sounds were clear and his abdomen was soft with a healthy midline scar of his previous surgery.

The blood results were as follows:

Haemoglobin 120 g l^−1^, (130–170 g l^−1^) white cell count 10.2 10*9 l^−1^ (4.0–11.0 × 10*9 l^−1^) , platelets 462 10*9 l^−1^ (150–400 10*9 l^−1^). The C-reactive protein (CRP) was elevated at 64 mg l^−1^ (0–9 mg l^−1^). His liver profile showed an elevated alanine transaminase level of 554 IU l^−1^ (0–35 IU l^−1^) and alkaline phosphatase 1446 IU l^−1^ (30–150 IU l^−1^), gamma-glutamyl transferase 670 IU l^−1^ (9–44 IU l^−1^), serum total bilirubin 9 umol l^−1^ (0–17 umol l^−1^), prothrombin time 11.1 s. (9.9–12.8 s), fibrinogen 8.10 g l^−1^ (2.00–5.10 g l^−1^), international normalized ratio 1.0 (0.9–1.2) and albumin 39 g l^−1^ (35–48 g l^−1^), serum amylase 70 IU l^−1^ (0–100 IU l^−1^), plasma glucose 7.1 mmol l^−1^ (4.0–7.7 mmol l^−1^). His corrected calcium was elevated at 2.76 mmol l^−1^ (2.15–2.60 mmol l^−1^) with a normal parathyroid hormone level of 3.6 pmol l^−1^ (1.9–6.4 pmol l^−1^). Electrolytes and kidney function test showed hyperkalaemia 6.7 mmol l^−1^ (3.5–5.0 mmol l^−1^) and mild hyponatraemia with a sodium level of 131 mmol l^−1^ (132–146 mmol l^−1^) with a kidney injury; creatinine 267 umol l^−1^ (59–104 umol l^−1^), urea 37.3 mmol l^−1^ (2.5–6.7 mmol l^−1^) and estimated glomerular filtration rate 21 ml min^−1^/1.73 m^−^^2^.

 Patient was initially managed with intravenous fluids along with optimization of his nutrition including nasogastric feeding with regular follow up by dieticians. A treatment with intravenous pamidronate was initiated to correct hypercalcaemia. A liver screen including a viral hepatitis screen was negative and a magnetic resonance cholangiopancreatography showed a dilated common bile duct and intrahepatic ducts. A prostate specific antigen was done to rule out possible prostate malignancy and bone metastasis. Myeloma screen was negative. Flexible sigmoidoscopy showed a benign looking polyp with no ulcers or any evidence of malignancy.

**Figure 1.**
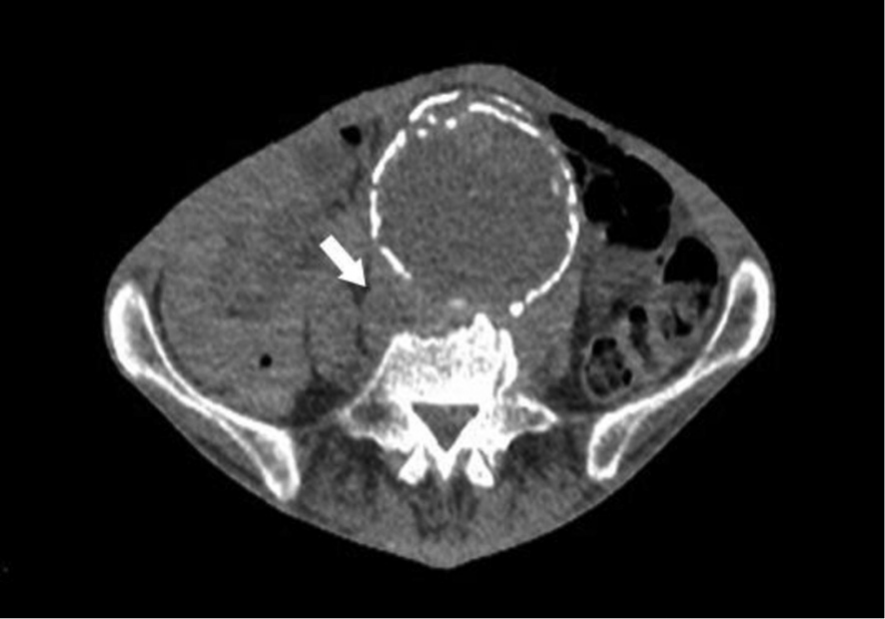
Non-contrast axial CT image showing an aneurysm with erosion of the anterior vertebral body and a soft tissue collection posterior to the aneurysm between the psoas muscle and the spine (arrow).

 An abdominal ultrasound showed normal kidneys and spleen structures and a thrombus in the aortic aneurysm. Later, CT chest, abdomen and pelvis showed soft tissue posterior to the aneurysm as well as periaortic inflammatory process, no malignancy was found (Figure 1). A diagnostic liver biopsy was considered to rule out possible liver pathologies that potentially explain the elevated liver enzymes but the patient declined owing to possible complications.

 After 6 weeks of admission, patient developed hospital-acquired pneumonia and was started on IV tazocin. His bloods showed elevated white blood cells (WBC) of 14.00 10*9 l^−1^, CRP of 199 mg l^−1^. Although his CRP was initially falling down in the first few days, patient’s back pain was progressive and his WBC and CRP climbed up markedly (20.7 10*9 dl^−1^, 154 mg l^−1^ respectively). Of note, patient did not develop high temperature—not once—during his hospital stay. A blood culture was done, one bottle showed positive growth of coagulase negative *Staphylococcus*. However, repeated blood culture after 48 h was negative.

**Figure 2.**
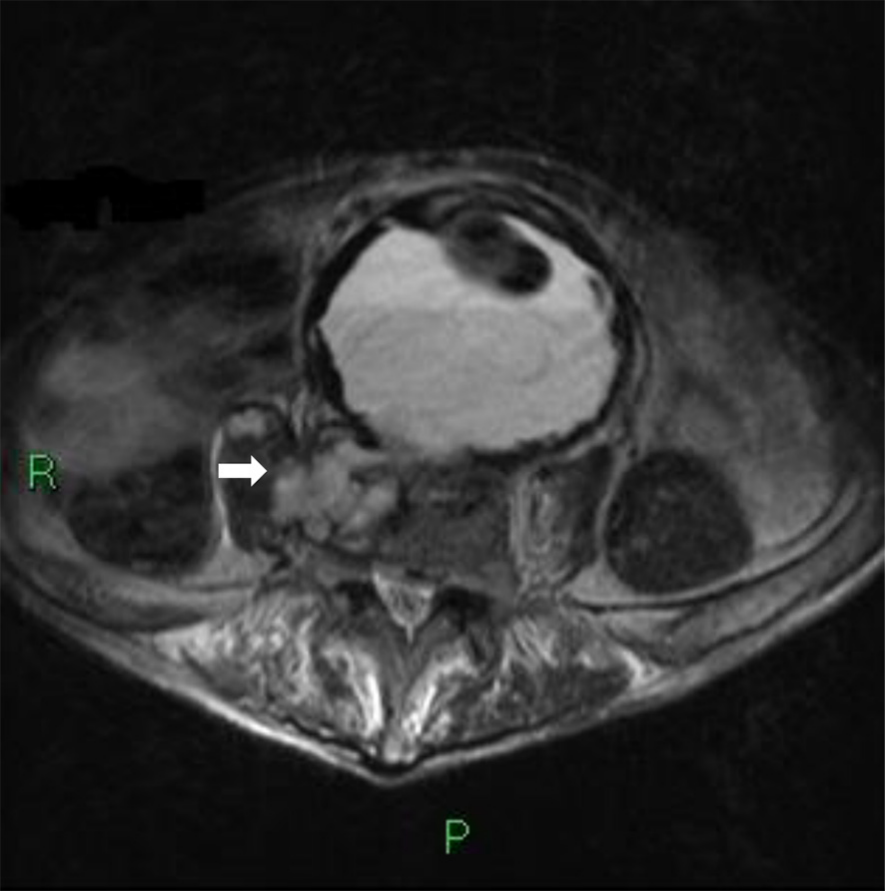
Axial *T*_2_ scan showing erosion of anterior L4 vertebra and extending collection in the form a small abscess over the right paravertebral region within the right psoas muscle (arrow).

 An MRI (lumbar spine) showed a small abscess collection over the right paravertebral region (Figure 2). Radiological and clinical diagnosis was one of an infected aortic graft and based on microbiology team advice, he was commenced on IV teicoplanin with the addition of rifampicin. Vascular team advised against surgical intervention, whereas neurosurgical team advised continuing intravenous antibiotic treatment and mobilization using Boston brace as able. Patient’s general condition was improving gradually; his mobility—on his chair—improved markedly and his infection markers declined on treatment, which was promising to allow for a plan to discharge the patient on long-term oral antibiotics when physiotherapy are happy.

**Figure 3.**
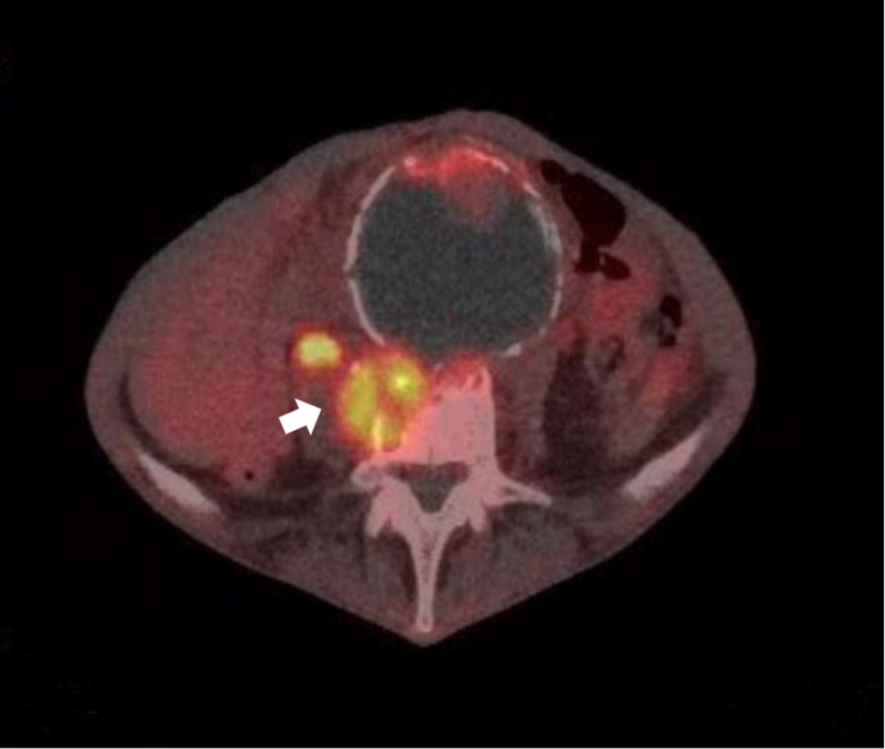
FDG-PET scan showing L4/5 disc destruction and intense FDG uptake within the disc with increased activity in the area of extension to the right psoas muscle (arrow). FDG, fludeoxyglucose.

 However, follow-up CT (abdomen/pelvis) was done after 10 weeks of antibiotic treatment and showed no improvement of previous findings. Moreover, an FDG-PET scan of lumbar spine showed an increased uptake in L4/5 disc with continuity to the right psoas muscle (Figure 3).

 Soon patient’s condition worsened with a progressive back pain rendering him bedridden. He developed low body temperature of 34.8°C and his WBC and CRP reached (17.1 10*9 l^−1^, 73 mg l^−1^ respectively). A repeated blood culture was negative. Owing to the progressive decline in the patient’s general status, despite a long antibiotic course of 15 weeks, he was commenced on an end of life care and died in early July 2017.

## Imaging findings

 Initial CT chest, abdomen and pelvis showed bony erosions of L4/5 with soft tissue posterior to the aneurysm between the psoas muscle and the spine (Figure 1), a follow-up CT scan showed no change in findings.

**Figure 4.**
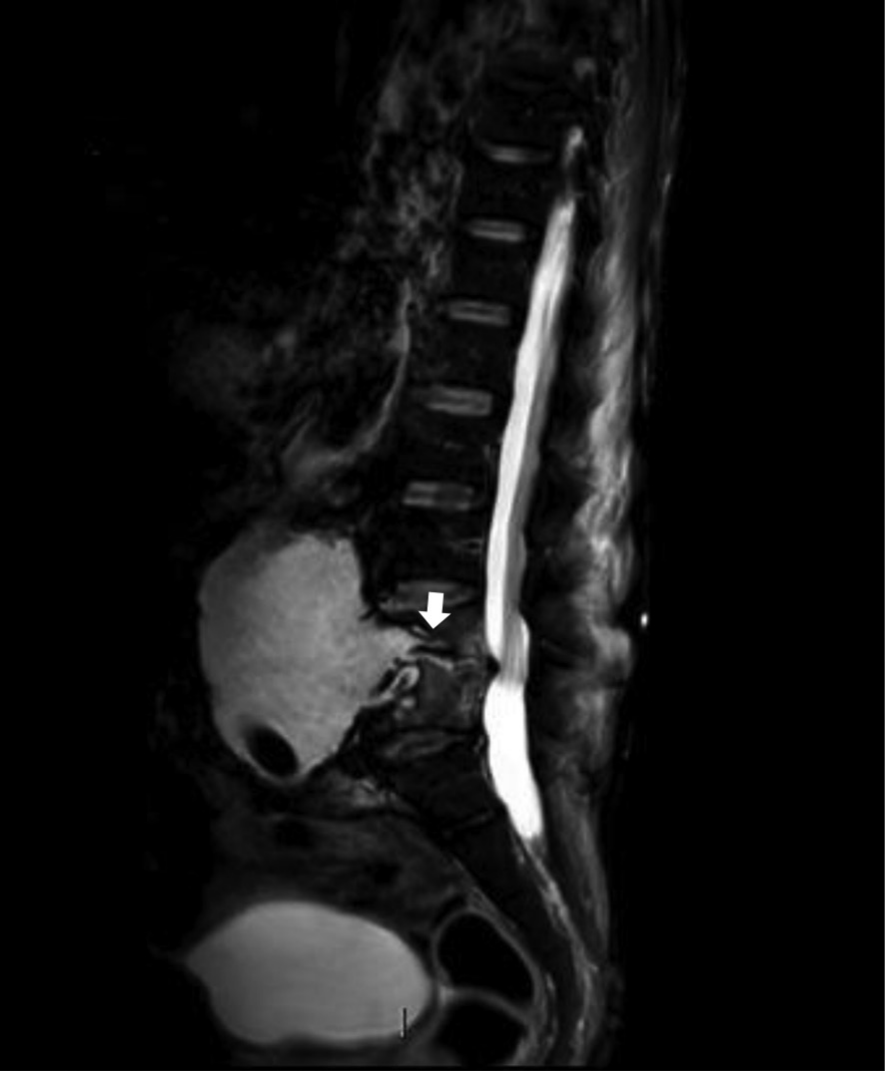
Sagittal MRI STIR showing communication of aneurysmal sac with L4/5 disc (arrow) with endplate destruction. STIR, short tau inversion-recovery.

**Figure 5.**
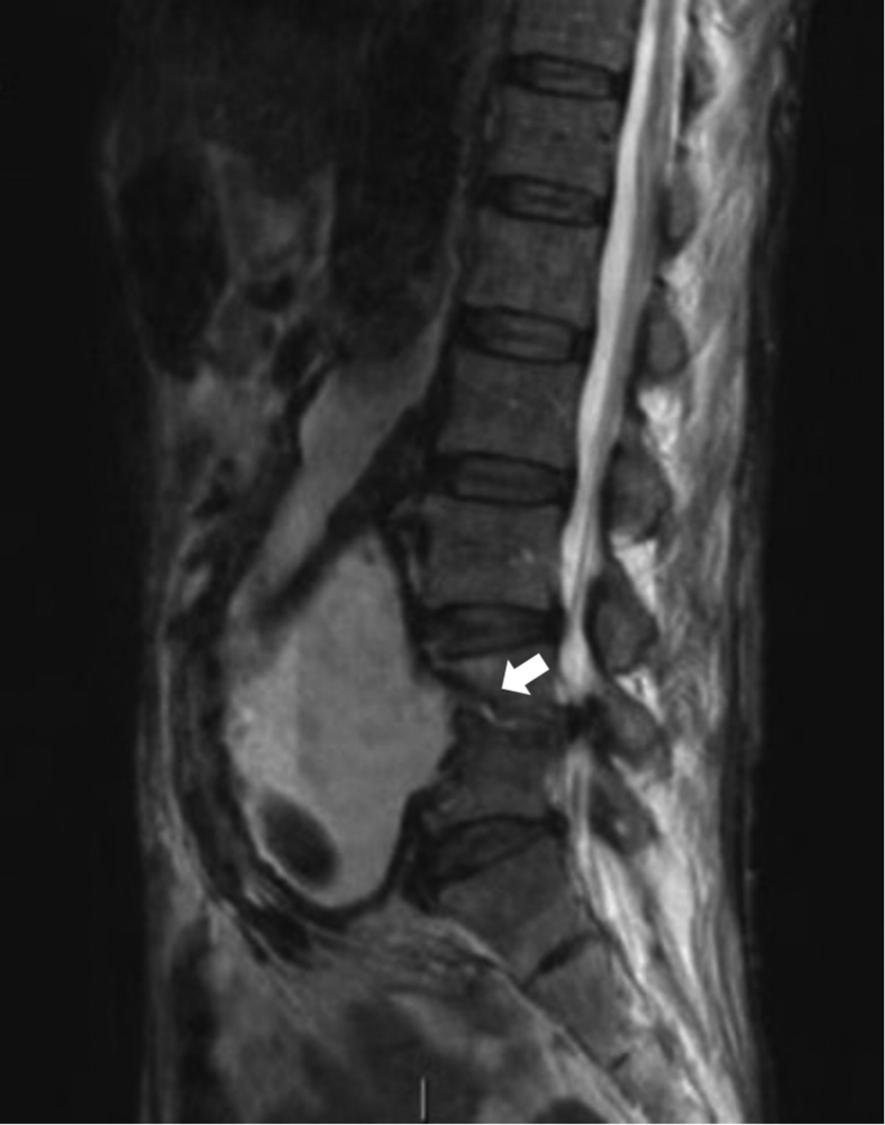
Sagittal *T*_2_ image showing fluid signal in the disc space L4\5 with loss of height, destruction of the endplate and communication between the aneurysmal sac and L4\5 disc (arrow).

 MRI lumbar spine showed fluid signal in the disc space L4\5 with loss of height, destruction of the endplate and extending collection in the form a small abscess over the right paravertebral region within the right psoas muscle along with a communication between the aneurysmal sac and L4\5 disc (Figures 2, 4 and 5).

FDG-PET scan showed intense FDG uptake within the L4/5 disc with a continuity with increased activity extending both superiorly and inferiorly along the anterior and medial aspect of the right psoas muscle along with increased uptake within the aneurysmal sac (Figure 3).

## Outcome

 Sadly, he presented severely cachectic with acute chronic renal failure, and despite the concerted efforts to manage his condition conservatively, he declined and died.

## Discussion

 Open surgical aortic grafting is one of the modalities used for the treatment of abdominal aortic aneurysms. One of the significant complications following surgery is the infection of the graft and/or the aneurysmal wall. Although, graft infection following aortic aneurysm repair is uncommon^1^ and continuous spread of infection to the spine is rare,^2, 3^ it needs prompt and urgent management as it is threatening and may lead to graft/arterial interface disruption, haemorrhage, or sepsis.^4^

 For a proper diagnosis, it is mandatory to do clinical examination, blood tests (CRP, WBC counts) and radiological investigations. As cultures of blood or even samples collected from the infected field are sometimes negative (up to 33% of cases),^4^ in those circumstances, diagnosis of graft infections are ultimately based on clinical and radiological evidence. Clinical manifestation of aortic graft infection with discitis is variable and lack specificity.^5^ Some *in vitro* reports have suggested that antimicrobial coated vascular grafts may protect against infection by organisms which can perioperatively colonize the vascular graft, but may not cause clinical findings of infection until a long time after,^6^ which could be the reason of the late presentation of our patient. Low-grade infection, which occurs in one-third of total patients (chronic sepsis)^4^ is caused by low-virulent bacteria and occurs generally late after the original graft implantation. Usually, these patients suffer from non-specific symptoms such as weakness, weight loss, and malaise; potentially leading to delayed diagnosis. Our patient had non-specific symptoms related to ongoing low-grade infection. Owing to the fact that his blood cultures were negative on two separate occasions and patient refused sampling from the infection field, it was mandatory to ascertain the diagnosis using different radiological investigations.

 CT scan is the most commonly used modality for the diagnosis of aortic graft/aneurysmal wall infections. Potential signs of graft infection on CT scan are perigraft fluid, perigraft soft tissue attenuation, ectopic gas and pseudoaneurysm. Yet, perigraft fluid and soft tissue attenuation are not specific for graft infection, as they might arise from chronic postoperative inflammatory process. Therefore, MRI scan has an additional value of a more accurate evaluation of soft tissue changes.

 FDG-PET scan is nowadays commonly used to diagnose various infections. Fukuchi et al described a high specificity of FDG-PET in the diagnosis of aortic graft infections when certain criteria of FDG-uptake pattern is met.^7^ A grading system was adopted for PDG-uptake, the intensity of which was graded on a 5-point scale, as follows: Grade 0, FDG uptake similar to that in the background; Grade 1, low FDG uptake, comparable to that by inactive muscles and fat; Grade 2, moderate FDG uptake, clearly visible and distinctly higher than the uptake by inactive muscles and fat; Grade 3, strong FDG uptake, but distinctly less than the physiological uptake by the bladder; and Grade 4, very strong FDG uptake, comparable to the physiological urinary uptake by the bladder.^7^ In our case, patient showed a high density uptake in L4\5 disc, focal uptake in the anterior part of the aneurysm (Figure 5) and the paraspinal collection that would match Grade 4–5 in this system. The direct extension of the aneurysmal wall into the disc with the presence of the paraspinal collection seen in the MRI scan favours the diagnosis of infection rather than the mere presence of the postoperative changes in the area of interest.

 As *staphylococcus* *aureus* was found to be a common cause of infection; in 54.5% of the cases as reported by Ducasse et al,^8^ patient was treated with intravenous teicoplanin and rifampicin. The source of infection was unknown in this case although the intra- and post-operative embolectomy interventions are thought to have probably introduced graft infections.

 According to multiple studies, outcome markedly worsens when conservative management is used.^8–10^ Sharif et al described a 100% mortality rate in conservatively managed patients.^9^ Heyer et al reported 75%,^10^ Ducasse et al reported 36.4%.^8^ Our patient had acute on top of chronic renal failure, which worsened the prognosis. Unfortunately, he died after conservative treatment failed to control his infection.

## Learning points

1. Aortic aneurysm graft infections are rare and could be misdiagnosed, unless there is an early index of suspicion.
2. Presentation of infected aortic grafts varies and could be subtle with general non-specific symptoms which may delay initiation of proper treatment.
3. CT, MRI and PET-CT scan has an increasing role in the diagnosis of graft infections and should be considered early in the course as laboratory evidence of infection might not be clear enough to raise the alarm for a prompt antibiotic therapy. Therefore, it is mandatory to carefully check for any signs of discitis in the CT scan and if there is a suspicion, a spine MRI should be immediately recommended.

**Figure 6.**
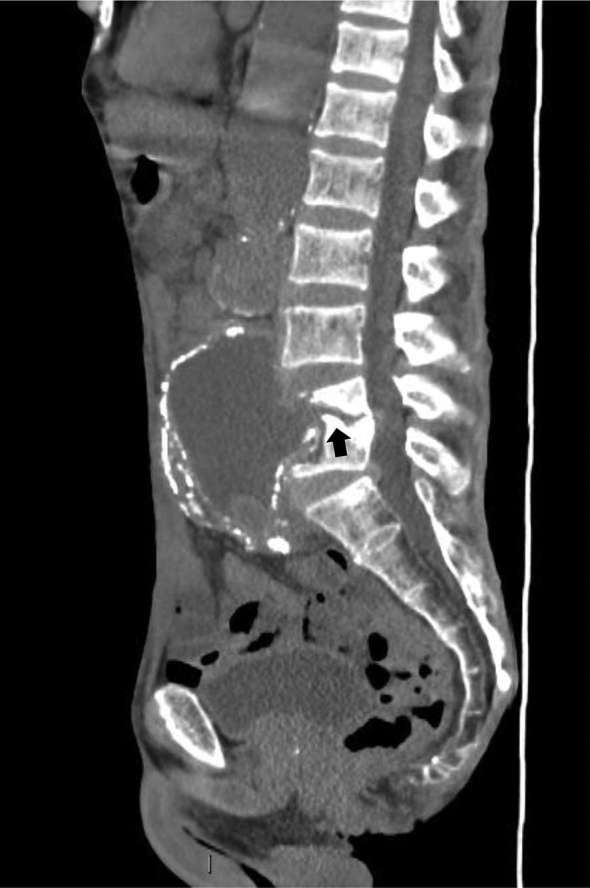
Sagittal CT showing communication of aneurysmal sac with L4/5 disc (arrow) with endplate destruction.
